# Utility of Ultraportable Echocardiography in the Preoperative Evaluation of Noncardiac Surgery

**DOI:** 10.5935/abc.20160169

**Published:** 2016-11

**Authors:** Jean Allan Costa, Maria Lucia Pereira Almeida, Tereza Cristina Duque Estrada, Guilherme Lobosco Werneck, Alexandre Marins Rocha, Maria Luiza Garcia Rosa, Mario Luiz Ribeiro, Claudio Tinoco Mesquita

**Affiliations:** 1 Programa de Pós-Graduação em Ciências Cardiovasculares da Universidade Federal do Rio de Janeiro, Niterói, RJ - Brazil; 2 Universidade Federal Fluminense - Hospital Universitário Antônio Pedro, Niterói, RJ - Brazil

**Keywords:** Echocardiography / methods, Diagnostic Imaging / methods, Preoperative Care, Elective Surgical Procedures

## Abstract

**Background:**

The ultraportable echocardiogram machine, with relevant portability and easiness in performing diagnoses, when in experienced hands, may contribute to the reliability of preoperative evaluation in noncardiac surgeries.

**Objectives:**

To assess cardiac function parameters in patients aged older than 60 years, candidates of elective noncardiac surgeries, classified as ASA1 or ASA 2 according to surgical risk.

**Methods:**

A total of 211 patients referred for elective surgeries, without suspicion of previous heart diseases, were included in the study. Assessment of patients was conducted by conventional echocardiogram using the ultraportable V Scan (GE) device right after the pre-anesthetic clinical evaluation. We assessed the clinical impact of echocardiography results by using a questionnaire addressed to the anesthetist.

**Results:**

Mean age of patients was 68.9 ± 7.0 years, 154 were women. The most frequent surgeries were: a) facectomy - cataract - 18; b) inguinal hernia surgery - 18; c) Cholecystectomy - 16. We found 58 normal tests (27.5%), 70 (33.2%) with mild valve reflux, and 83 (39.3%) with relevant abnormality, such as increase in heart chamber size, global and/or segmental contractile dysfunction, significant valve dysfunction or other unspecified. Test results caused delay of surgical procedure for a more detailed cardiac evaluation in 20 (9.5%) patients, and change in anesthetic management in 7 (3.3%).

**Conclusion:**

There was a considerable clinical impact with the use of the ultraportable echocardiography, since one out of every ten patients evaluated had their clinical management changed due to the detection of previously unsuspected, significant heart diseases, with the potential for severe complications.

## Introduction

Of all imaging tests, echocardiography is the most required test in the field of cardiovascular diagnosis.^1^ Transthoracic echocardiography corresponds to approximately 50% of all cardiovascular imaging tests ordered in the USA.^2^ The reasons of the success of echocardiography include its low cost, wide availability, absence of adverse effects or radiation exposure, and possibility to be performed at bedside. One of the most recent advances in miniaturization of ultrasound (US) devices is the ultraportable US, with high portability, easy handling, and good image quality. However, the ultraportable equipment does not allow evaluations in one-dimensional, pulsed Doppler or continuous Doppler modes, limiting the detection and dimensioning of some cardiac changes.

It is estimated that more than 230 million surgeries per year are performed worldwide.^3^ The most common perioperative cardiac complications are acute myocardial infarction, cardiac arrest and acute cardiac failure - affecting nearly 5% of patients aged 70 years or older undergoing noncardiac surgeries.^3^ In Brazil, according to the 2008 data of the Ministry of Health, approximately 3 million noncardiac surgeries are performed annually, with a cost of nearly 2.4 billion Brazilian reals and perioperative mortality rate around 2.3%.^4^

The indication of tests and cardioprotective or therapeutic measures for a patient who will undergo a noncardiac surgery ultimately results from the balance between the probability of recovery and risk.^5^ The routine use of resting echocardiography as a risk stratification tool is discouraged, and is considered a class III recommendation according to the II Guidelines on Perioperative Cardiovascular Evaluation of the Brazilian Society of Cardiology, 2011.^6^

The rationale of the present study was that the routine ordering of conventional echocardiography for individuals at higher risk, or even for those aged older than 60 years with chronic diseases may not be that useful in the preoperative evaluation. It could also delay the scheduling of surgical procedures and expose patients to risks of complications and even death before surgery. In this context, we tested the usefulness of the ultraportable device to detect unsuspected heart diseases in the routine preoperative evaluation and its impact on the clinical practice.

## Methods

Patients of both sexes, aged older than 60 years, referred to elective noncardiac surgeries in a tertiary hospital were included in the study. According to the American Society of Anaesthesiologists' classification, all patients were classified as ASA 1 or ASA 2 for surgical risk, without known or suspected previous heart disease. After pre-anesthetic evaluation, patients were assessed using the ultraportable US device, by following the same criteria of conventional echocardiography, except for the resources missing in the ultraportable device. Exclusion criterion was history of previous heart diseases. All participants signed the informed consent form. The study was conducted according to the principles established in the Helsinki declaration and revised in 2000 (Scotland, 2000). The study was approved by the ethics committee of Faculdade de Medicina da UFF. CEP HUAP (protocol: 13903213.5.0000.5243).

The tests were performed using the ultraportable Vscan US (GE Healthcare, Milwaukee, Wisconsin, EUA), by a cardiologist echocardiographer. For comparison of the reports, the images were stored and analyzed by another cardiologist echocardiographer, who was unaware of previous medical reports.

Subjective analyzes of two-dimensional images and color flow mapping were performed, focusing on the following aspects: dimensions of cardiac chambers, global and segmental left ventricular contractile function, right ventricular systolic contractile function, valve anatomy and movement, presence of valve reflux, indirect signs of pulmonary hypertension or increased central venous pressure, presence of pericardial effusion or thickening, and thoracic aorta dimensions.

Clinical usefulness of the technique was assessed by a three-question questionnaire aimed to clarify the importance of the echocardiography report for the decision-making process regarding the surgical procedure, and what changes from the initial plan have been made. In this questionnaire, the main information requested was:

1. With respect to the procedure, the echo report was considered:
  ( ) not important ( ) moderate importance
  ( ) decisive ( ) little important
  ( ) very important
2. Did the echo report make you feel more confident to perform the procedure?
  ( ) yes ( ) no
3. Did the echo report contribute to any change in the management of the patient who will undergo surgery?
  1 ( 0 ) no
  2 ( 0 ) I just became more alert
  3 ( 0 ) I increased the vigilance and monitoring of the patient
  4 ( 0 ) I changed the preparation of the patient
  5 ( 1 ) I changed the anesthetic management
  6 ( 2 ) I waited for a more complete cardiac assessment
  7 ( 2 ) I postponed it
  8 ( 2 ) I canceled it

0 = Anesthetic management and procedure scheduling maintained.

1 = Anesthetic management changed and procedure scheduling maintained.

2 = Scheduling changed.

The first question aimed to evaluate the importance given by the professional to the echo report to make his/her decisions. The second question, although quite obvious, gives an idea of the influence of the echo report on the taking of informed decisions. The third question defines the outcome of the management with respect to the patient and the procedure proposed, based on the echocardiographic findings. Thereby, we considered the third question the one that defines the course of the clinical management after being aware of the findings obtained with the ultraportable device. We considered the items 1-4 as less important ones, as they did not affect relevant technical data related to the procedure, such as anesthetic technique or the date of the procedure. Item 5 was considered as a mild outcome, since it was sufficiently important to change the anesthetic technique, while maintaining the procedure scheduling. Items 6-8 were considered less important outcomes, as the data changed the date of the procedure. In all cases, a more complete cardiac evaluation was awaited.

Therefore, we considered three possible results to occur:

**Group 1** - Patients that had their clinical / anesthetic management and schedule maintained.

**Group 2** - Patient that had their clinical / anesthetic management and schedule significantly altered, without affecting the procedure scheduling (change of anesthetic agent, hydration or support drugs).

**Group 3** - Patients that had their procedure schedule changed, such as a delay (for a better cardiovascular evaluation) or canceling of the planned procedure.

Regarding group 2, as change in patients' clinical / anesthetic management, we considered a change in the anesthetic technique to reduce the effects of the anesthetic agents on the cardiovascular system, or a change in associated drugs to reduce the risk of complications. In the third question, the anesthetist could check more than one answer, provided that the answers were not contradictory.

### Statistical analysis

By descriptive analysis, data were shown in tables; categorical data were expressed as frequency (n) and percentage (%), whereas numerical data as mean and standard deviation. Inferential analysis was composed by kappa coefficient to evaluate the inter-observer (or inter-method) agreement in the classification of cardiologic parameters. The 5% level was used as criterion for significance. Statistical analysis was performed by the SPSS software, version 20.0.

## Results

From April 2013 to June 2011, we studied 211 patients (73% women) aged ≥ 60 years (68.9 ± 7.0 years) referred to elective noncardiac surgeries, previously evaluated for preoperative and preanesthetic risks, and classified as ASA-1 or ASA-2 for surgical risk. Most of them were white (68.7%), and the mean body mass index was 28.2 kg/m^2^ (Table 1).

**Table 1:** Characteristics of the study population

Characteristics	Nº	%
**Total**	211	100%
**Sex**		
Men	57	(31.3)
Women	154	(73.0)
**Skin color**		
White	145	(68.7)
Brown or black	66	(28.7)
**Surgery size**		
Minor	87	(41.2)
Intermediate	112	(53.1)
Major	12	(5.7)
**Surgical risk**		
ASA 1	8	(3.8)
ASA 2	203	(96.2)
**Diabetes Mellitus II**		
Yes	47	(22.3)
No	164	(77.7)
**Hypertension**		
Yes	126	(67.0)
	**Mean**	**(SD)**
**Age**	68.9	(7.0)
**Systolic arterial pressure**	146.8	(25.4)
**Diastolic arterial pressure**	83.5	(11.4)
**Heart rate**	78.4	(5.8)
**Body mass index**	28.2	(5.7)
**Weight (Kg)**	70	(15)
**Height (cm)**	158	(10)
**Abdominal circumference (cm)**	102	(10)
**BSA**	1.711	(0.199)

ASA: American Society of Anesthesiology; BSA: body surface area.

The most frequent surgeries were: facectomy (cataract) (n=18) and inguinal hernia surgery (n=18), and cholecystectomy (n=16). A total of 58 tests (27.5%) were considered normal, 70 (33.2%) with mild valve regurgitation, and 83 (39.3%) with some relevant abnormality such as increase in heart chamber size, global and/or segmental contractile dysfunction, significant valve dysfunction or other unspecified.

In tables 2 and 3, we listed the electrocardiogram (ECG) and chest X-ray reports provided by the respective divisions (cardiology and radiology), pointing out the most frequent changes.

**Table 2:** Frequency of changes found in preoperative electrocardiogram reports (modified)

Electrocardiogram results	Number of patients	%
Normal	83	(39.3)
Nonspecific ventricular repolarization changes	49	(23.2)
Chamber overload	12	(5.7)
Ischemic	6	(2.8)
Others	20	(9.5)
Without the test	41	(19.4)
Total	211	(100)

**Table 3:** Frequency of changes found in chest X-ray reports (modified)

Chest X-ray results	Number of patients	%
Normal	122	(57.8)
Cardiac area at the upper limits of normal	7	(3.3)
Increased cardiac area	8	(3.8)
Impaired evaluation of cardiac area	3	(1.4)
Others	1	(0.5)
Without the test	70	(33.2)
Total	211	(100)

On table 4, it is clear that there was no significant association between ECG reports and changes detected by the ultraportable US.

**Table 4:** Comparison of normal electrocardiograms (total = 83) with changes detected by the ultraportable electrocardiogram machine

LV hypertrophy	1	1
LV dimensions	Slight increase	4
LA	Slight increase	16
LA	Moderate increase	1
RV	0	0
RA	Slight increase	1
GVF	Slight decrease	1
SEGVF	Hypokinesia	2
SEGVF	Hypocinesia and akinesia	1

LV: left ventricle; LA: Left atrium; VD: right ventricle; RA: right atrium; GVF: global ventricular function; SEGVF: segmental ventricular function.

Of the 8 patients with increased cardiac area at chest X-ray, 2 patients had normal echocardiography, and 4 had a slight increase in any of the cardiac chambers. On the other hand, of 122 patients with normal X-ray, 29 had a slight increase of left atrium (LA), 5 had a slight increase of left ventricle (LV), 8 had left ventricular hypertrophy, 4 had a moderate increase of LA and 2 had an important increase of this chamber.

In the analysis of ECG, we found that of 83 patients with normal ECG, 16 patients had slight increase of LA dimensions, 4 had slight increase of LV, 1 had slightly depressed left ventricular global systolic function, and 3 had segmental contractile dysfunction.

### Echocardiographic evaluation:

In all patients the with the ultra portable echocardiography test could be performed with the following findings: 58 patients had normal result, 70 patients had mild valve reflux, and 83 had other abnormalities, which were more expressive than mild valve reflux.

The most prevalent echocardiographic abnormalities were:

1. Increase of LA - 45 patients (25%),
2. Changes in segmental contractility - 12 patients (6%),
3. Left ventricular hypertrophy - 9 patients (5%).

Other abnormalities included: 5 patients with slight increase of left ventricular diameters, altered global systolic function in 4 patients (3 with mild dysfunction and 1 with moderate dysfunction), and 12 with altered segmental contractility. We observed an increase of the right atrium in 7 patients, 3 of them with relevant increase, and 3 with moderate increase.

Echocardiographic evaluation of valve function revealed narrowed mitral valve opening in 4 patients, 2 of them with mild narrowing, 1 with moderate and 1 with important narrowing. Narrowing of the aortic valve opening was observed in 21 patients, 15 of them classified as mild, 5 as moderate and 1 as important. Mitral insufficiency was prevalent in 48.8% of patients or 103 cases, 96 of them with mild insufficiency, 5 with moderate insufficiency and 2 with important insufficiency. Aortic insufficiency was observed in 61 patients (32.5%), 58 of them with mild insufficiency and 3 with moderate insufficiency. Tricuspid regurgitation occurred in 49 patients, 42 of them with mild insufficiency, 5 with moderate and 2 with important insufficiency.

Other abnormalities were: 6 cases of mild thoracic aortic dilatation and 1 case of moderate dilatation. Evaluation of the pericardium revealed 5 cases of mild increase of pericardial fluid.

Reports of the US performed with the ultraportable device were sent to a professional of the anesthetic department of Antônio Pedro University Hospital. The opinion of this professional was expressed in the questionnaire, which was decisive for the final direction of patient's treatment.

The anesthetist's questionnaire answers are described in table 5, which expresses the relationship between changes detected by the ultraportable US device and the decisions made by the anesthetist toward the procedure to be performed on the patient. In 20 patients, the schedule of the surgical procedure was changed, and in 7 patients, the clinical management was changed without changing the schedule. By comparing these data with the historical mean (5%) of procedures that have suffered changes following immediate preoperative anesthetic evaluation, we found a significant association of the use of the ultraportable echocardiography with changes in patient's management, with an odds ratio of 2.9 (p = 0.003, 95% confidence interval of 1.39-6.26).

**Table 5:** Frequency of the anesthetist’s answers and anesthetic management following the echocardiographic report

			Clinical management and schedule maintained	Clinical management changed and schedule maintained	Schedule changed
Total	211	100	184 (87.2)	7 (3.3)	20 (9.5)
	Nº	**%**			
I just became more alert	74	35.1	60	0	0
I increased the vigilance and monitoring of the patient	96	45.5	91	0	0
I just became more alert and increased the vigilance and monitoring of the patient	10	4.7	4	0	0
I changed the preparation of the patient	3	1.4	3	0	0
I changed the anesthetic management	1	0.5	0	2	0
I changed the preparation of the patient and the anesthetic management	2	0.9	0	1	0
I changed the anesthetic management and increased the vigilance and monitoring of the patient	5	2.4	0	5	0
I waited for a more complete cardiac evaluation	9	4.3	0	0	9
I postponed the procedure and waited for a more complete cardiac evaluation	10	4.7	0	0	10
M I changed the preparation of the patient, postponed the procedure and increased the vigilance and monitoring of the patient	1	0.5	0	0	1

There was a good inter-observer agreement regarding analysis of the V Scan images, except for the analysis of left ventricular dimensions and its segmental function, which was shown to be satisfactory (Table 6). Comparisons with tests performed with conventional echocardiography device (iE Philips) were limited by the small number of cases.

**Table 6:** Inter-observer agreement analysis of cardiac parameters evaluated by cardiac ultrasound

Cardiac parameters		Changed by VSCAN1		VSCAN1 x VSCAN2		
		N	%	Kappa	SE	p value
1	Left ventricular dimension (hypertrophy)	10	4.7	0.69	0.12	< 0.001
2	Left ventricular dimension	7	3.3	0.29	0.17	< 0.001
3	Left atrial dimension	53	25.1	0.51	0.07	< 0.001
4	Right atrial dimension	7	3.3	0.53	0.18	< 0.001
5	Global ventricular function	6	2.8	0.80	0.14	< 0.001
6	Segmental ventricular function	14	6.6	0.38	0.13	< 0.001
7	Mitral valve (leaflet mobility)	4	1.9	0.86	0.143	< 0.001
8	Mitral valve (regurgitation)	114	54.0	0.52	0.06	< 0.001
9	Aortic valve (leaflet mobility)	25	11.8	0.51	0.10	< 0.001
10	Aortic valve (regurgitation)	66	31.3	0.49	0.06	< 0.001
11	Tricuspid valve (regurgitation)	55	26.1	0.56	0.063	< 0.001

SE: standard error.

In this context, we highlight the importance of the changes detected by the ultraportable ultrasound device, which was crucial to indicate unsuspected cardiac changes that could represent perioperative complications.

## Discussion

Cardiac ultrasound with the ultraportable device is a new tool that has being increasingly used as a complementary test to clinical examination, due to its rapid execution, prompt availability, at relatively low cost. It has been recently demonstrated that this technology has an incremental diagnostic value to cardiologic clinical examination, by increasing the number of correct diagnosis and reducing the routine use of echocardiography.^7^

In our study, we found that the ultraportable machine was useful to detect clinically important heart diseases in patients previously approved for the surgery, with ASA surgery risk 1 or 2. A significant percentage of previously unknown cardiac abnormalities have been identified, and in some cases, the surgery had to be delayed for a better cardiovascular assessment. Today the physician has limited amount of time to see the patient and perform a thorough examination including anamnesis, physical examination, ordering of exams, making notes on patients' medical records, issue of opinions and reports, drug prescription, referral of patient to other services, explanation about the health problem, and the importance of correct use of the drug prescribed, consequences of its incorrect use and possible side effects to the patient. In many cases, when associated with an unfavorable biotype, these factors may hamper the detection of important heart disease by clinical evaluation.^8-17^ As shown in this study, changes detected by routine complementary exams normally ordered in the preoperative evaluation do not have a linear relationship with the presence of significant heart diseases, which make the identification of these diseases even more difficult. Consequently, a considerable number of patients aged older than 60 years may have an important heart disease, and this fact unknown by the patient or even by his physician.

The use of ultraportable echocardiography has been an object of study in several areas of cardiology, and recently been focused in the preoperative evaluation. Cavallari and colleagues published a study on the use of ultraportable cardiac US in patients with indication of noncardiac surgery.^18^ The authors randomly assessed 100 patients with known heart disease or important cardiovascular complaints referred to echocardiography by a cardiologist. By comparing the use of the ultraportable cardiac US (Opti-Go Philips) with the conventional echocardiography (Philips iE33), the investigators found the same rate of diagnostic conclusiveness between the techniques, although the former is faster and promotes a less waiting time, which optimizes the preoperative evaluation.^18^ In this study, 211 patients without suspicion of heart diseases and previously approved for surgeries were studied, which may explain the higher percentages of tests without significant changes in our sample (60.7% versus 12.8%). In the Italian study,^18^ patients were randomized to one of two techniques, and 50 patients underwent the ultraportable cardiac US, whereas in our study, all patients underwent this technique.

Several studies have pointed out the importance of the ultraportable US in reducing the time and in its accuracy in detecting or confirming heart diseases in patients with suspected or even confirmed previous heart diseases.^19-28^ Our study emphasizes the use of the new technique in the detection of unsuspected heart diseases, particularly in patients at risk, as those already approved for a noncardiac surgery. These data highlight the importance of the new technique in improving the search for heart diseases in populations without suspicion of previous diseases, but with risk factors for these abnormalities.

## Conclusion

The ultraportable US device is a viable tool, easily implemented in the preoperative evaluation. In our study, the most frequent abnormalities detected by the ultraportable echocardiography were mild valve reflux and increased chamber sizes. There was a considerable clinical impact of its use, since out of every ten patients investigated, one required suspension of the surgery for a more extensive cardiovascular evaluation. Also, we may infer that a significant number of individuals with undertreated or untreated chronic diseases, important heart diseases and potential for severe complications, may benefit from the use of this new technology. Although ECG and chest X-ray tests continue to be important tools in the preoperative evaluation, the use of the ultraportable echocardiography was decisive for the detection of cardiac abnormalities not identified by these techniques due to inherent limitations.

## References

[1] Patel MR, Peterson ED, Dai D, Brennan JM, Redberg RF, Anderson HV (2010). Low diagnostic yield of elective coronary
angiography. N Engl J Med.

[2] Barbosa FC, Mesquita ET, Salgado A, Mesquita CT (2010). Quality in Cardiac Imaging: appropriateness criteria applied to
echocardiography. Rev Bras Cardiol.

[3] Yu PC (2010). Registro nacional de operações não
cardíacas: aspectos clínicos, cirúrgicos,
epidemiológicos e econômicos.

[4] Ministério da Saúde Secretaria executiva DATASUS-SUS.

[5] Buitrago FJ, Santana JA, Guimarães LF, Henriques MD, Almeida WM (2011). Avaliação cardiovascular perioperatória para
cirurgia não cardíaca. REv Med Minas Gerais.

[6] Gualandro DM, Yu PC, Calderaro D, Marques AC, Pinho C, Caramelli B (2011). II Guidelines for Perioperative Evaluation of the Brazilian
Society of Cardiology. Arq Bras Cardiol.

[7] Mancuso FJ, Siqueira VN, Moisés VA, Gois AF, Paola AA, Carvalho AC (2014). Focused cardiac ultrasound using a pocket-size device in the
emergency room. Arq Bras Cardiol.

[8] Benson S, Vance-Bryan K, Raddatz J (2000). Time to patient discontinuation of antihypertensive drugs in
different classes. Am J Health Syst Pharm.

[9] Teixeira JJ, Lefévre F (2001). Drug prescription from the perspective of elderly
patients. Rev Saúde Pública.

[10] Mion D, Machado CA, Gomes MA, Nobre F, Kohlmann O, Amodeo C, Sociedade Brasileira de Cardiologia, Sociedade Brasileira de Hipertensão, Sociedade Brasileira de Nefrologia (2004). IV Diretrizes brasileiras de hipertensão
arterial. Arq Bras Cardiol.

[11] Rosenfeld S (2003). Cad Saúde. Pública.

[12] Chaimowicz F (1998). Os idosos brasileiros no século XXI:demografia, saúde e
sociedade.

[13] Veras RP (2003). Em busca de uma assistência adequada à saúde
do idoso: revisão de literatura e aplicação de um
instrumento de detecção precoce e de previsibilidade de
agravos. Cad Saúde Pública.

[14] Cesarino CB (2000). Eficácia da educação conscientizadora no controle
da hipertensão arterial sistêmica.

[15] da Silva T, Dall-Pizzol F, Bello CM, Mengue SS, Schenkel EP, Drug package inserts and the adequacy of patient's drug
information (2000). Rev Saúde. Publica.

[16] (2003). Pharmacy-nursing shared vision for safe medication use in
hospitals: executive summary session. Am J Health Syst Pharm.

[17] Borestein JE, Graber G, Salatiel E, Wallace J, Ryu S, Archi J (2003). Physician-pharmacist co-management of hypertension: a randomized,
comparative trial. Pharmacotherapy.

[18] Cavallari I, Mega S, Goffredo C, Patti G, Chello M, Di Sciascio G (2015). Hand-held echocardiography in the setting of pre-operative
cardiac evaluation of patients undergoing non-cardiac surgery: results from
a randomized pilot study. Int J Cardiovasc Imaging.

[19] Marques AC, Caramelli B (2008). Echocardiogram in the operative setting. Rev Bras Ecocardiogr.

[20] Roelandt JR (2004). Ultrasound stethoscopy. Eur J Intern Med.

[21] Roelandt J, Wladimiroff JW, Baars AM (1978). Ultrasonic real time imaging with a handheld-scanner. Part
II-initial clinical experience. Ultrasound Med Biol.

[22] Prinz C, Voigt JU (2011). Diagnostic accuracy of a hand-held ultrasound scanner in routine
patients referred for echocardiography. J Am Soc Echocardiogr.

[23] Cardim N, Fernandez Golfin C, Ferreira D, Aubele A, Toste J, Cobos MA (2011). Usefulness of a new miniaturized echocardiographic system in
outpatient cardiology consultations as an extension of physical
examination. J Am Soc Echocardiogr.

[24] Kimura BJ, Gilcrease GW, Showalter BK, Phan JN, Wolfson T (2012). Diagnostic performance of a pocket-sized ultrasound device for
quick-look cardiac imaging. Am J Emerg Med.

[25] Andersen GN, Haugen BO, Graven T, Salvesen O, Mjølstad OC, Dalen H (2011). Feasibility and reliability of point-of care pocket-sized
echocardiography. Eur J Echocardiogr.

[26] Sicari R, Galderisi M, Voigt JU, Habib G, Zamorano JL, Lancellotti P (2011). The use of pocket-size imaging devices: a position statement of
the European Association of Echocardiography. Eur J Echocardiogr.

[27] Galderisi M, Santoro A, Versiero M, Lomoriello VS, Esposito R, Raia R (2010). Improved cardiovascular diagnostic accuracy by pocket size
imaging device in non-cardiologic outpatients: the NaUSiCa (Naples
Ultrasound Stethoscope in Cardiology) study. Cardiovasc Ultrasound.

[28] Egan M, Ionescu A (2008). The pocket echocardiograph: a useful new tool?. Eur J Echocardiogr.

